# A case with bilateral C-shaped autofluorescence in retinal degeneration

**DOI:** 10.1016/j.ajoc.2025.102351

**Published:** 2025-05-02

**Authors:** Hsin Hsu, Chunya Kang, Eugene Yu-Chuan Kang, Nan-Kai Wang

**Affiliations:** a Department of Medicine, College of Medicine, Chang Gung University, Taoyuan, Taiwan; b Department of Education, Keelung Chang Gung Memorial Hospital, Keelung, Taiwan; c Department of Ophthalmology, Chang Gung Memorial Hospital, Linkou Medical Center, Taoyuan, Taiwan; d Department of Ophthalmology, Vagelos College of Physicians and Surgeons, Columbia University Irving Medical Center, New York, NY, USA; e Graduate Institute of Clinical Medical Sciences, College of Medicine, Chang Gung University, Taoyuan, Taiwan

**Keywords:** *SSBP1*, OPA13, Optic atrophy, Fundus autofluorescence

## Abstract

**Purpose:**

To report a case of a 19-year-old male with Optic Atrophy-13 (OPA13) associated with a de novo heterozygous missense variant in the single-strand DNA-binding protein 1 (*SSBP1*) gene, characterized by a bilateral double C-shaped hyper-autofluorescent ring on fundus autofluorescence (FAF).

**Observations:**

A 19-year-old male exhibited poor visual acuity, pale optic discs, vessel attenuation, and peripheral pigmentary changes. FAF imaging revealed a bilateral double C-shaped hyper-autofluorescent ring, which was not frequently observed. Optical coherence tomography (OCT) showed retinal thinning and ellipsoid zone disruption, while electroretinography (ERG) indicated cone-rod dystrophy. Genetic testing identified a pathogenic *SSBP1* missense variant, confirming the diagnosis of OPA13. Parental genetic analysis excluded the variant, establishing it as a de novo one.

**Conclusions and importance:**

This report highlights a novel retinal feature—a double C-shaped autofluorescent ring—associated with OPA13, potentially serving as a diagnostic marker for the disease. The findings emphasize the role of genetic testing in diagnosing OPA13 and distinguishing it from other retinal and optic neuropathies. Recognition of this feature could enhance early diagnosis and management strategies for patients with suspected OPA13.

## 1 Introduction

The single-strand DNA-binding protein 1 gene (*SSBP1*, MIM: 600439) is a housekeeping gene essential for mitochondrial biogenesis.^1^ *In vitro*, the *SSBP1* protein is required for mitochondrial DNA (mtDNA) copy number, playing a vital role in mtDNA replication and repair.^2^ Variants in the *SSBP1* gene can alter *SSBP1* protein levels and structure, hindering proper mtDNA repair and leading to mitochondrial dysfunction and associated diseases.^2^

Optic atrophy-13 (OPA13), an autosomal dominant disorder associated with retinal and foveal abnormalities, is characterized by reduced visual acuity due to bilateral optic atrophy and often includes foveopathy.^2^^,^^3^ This disease is caused by variants in the *SSBP1* gene. Studies in 2019 and 2020 documented families with OPA13 linked to heterozygous *SSBP1* variants, further substantiating the association between OPA13 and *SSBP1* variants.^2^^,^^4^ These variants may impair replication machinery in retinal ganglion cells (RGCs) and other cell types.^2^^,^^3^ A total of 47 cases of retinopathy associated with mutant *SSBP1* have been reported to date. In the study by Del Dotto et al. (2019), retinopathy was described in 8 individuals from 5 families.^2^ Piro-Megy et al. (2019) reported 14 cases, while Jurkute et al. (2019) identified 12 patients with retinopathy linked to the *SSBP1* variant.^3^^,^^4^ Finally, Meunier et al. (2021) described 12 cases, and Chang et al. (2023) reported 1 additional case.^5^^,^^6^ Here, we present a 19-year-old male with OPA13, showing a double C-shaped hyperfluorescent ring in fundus autofluorescence (FAF), which has not been reported and needs further investigation.

## 2 Case report

**Fig. 1:**
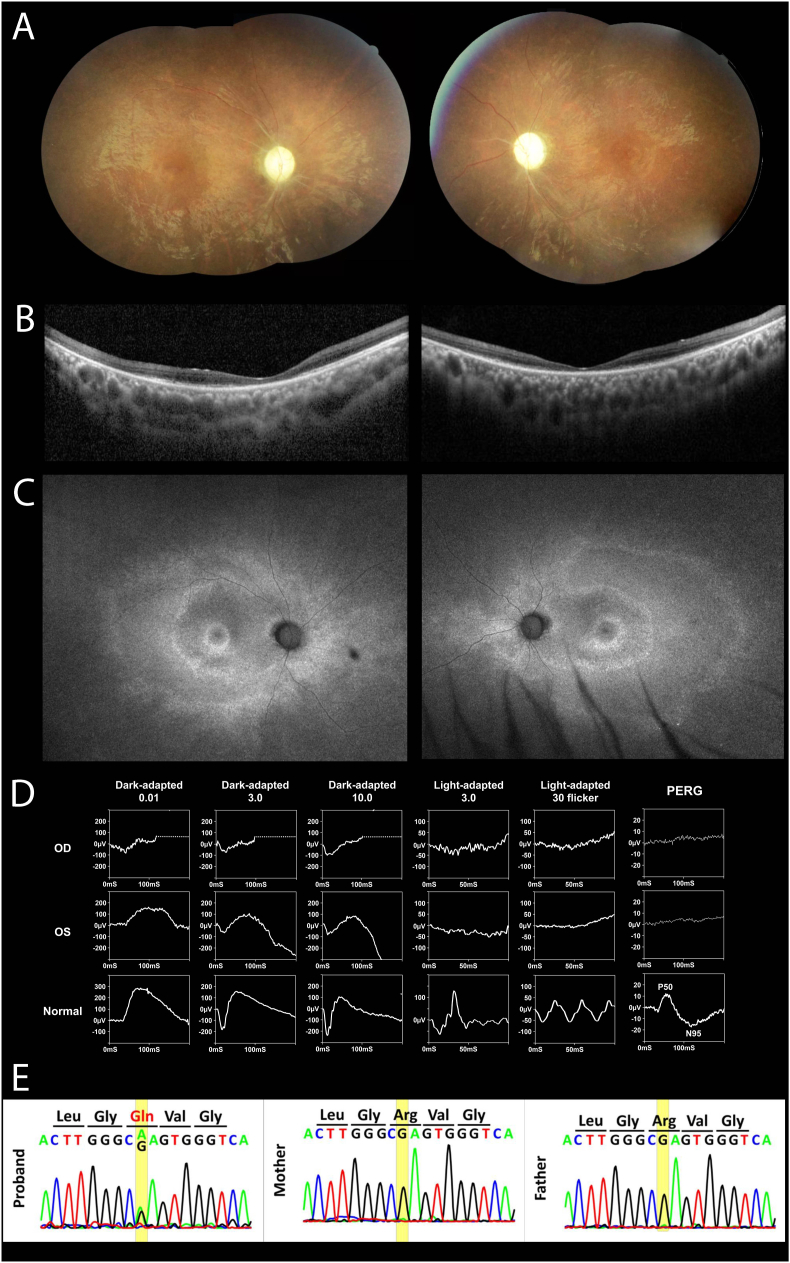
**(**A) Color fundus photography shows vessel attenuation, papillomacular bundle loss, and peripheral pigmentary changes. (B) OCT reveals retinal thinning, ellipsoid zone disruption, and intraretinal cysts. (C) Full-field ERG reveals reduced rod response and absent cone response. (D) FAF displays a C-shaped pattern with a double hyperfluorescent ring in both eyes. (E) Sanger sequencing identifies the *SSBP1* variant c.113G > A (p.Arg38Gln) in the patient, with negative results for both parents OCT, Optical coherence tomography; *SSBP1*, single-strand DNA-binding protein 1; ERG, electroretinogram; FAF, Fundus autofluorescence. (For interpretation of the references to colour in this figure legend, the reader is referred to the Web version of this article.)

In this case, a 19-year-old male with poor vision since early childhood has a medical history of amblyopia, premature ventricular contractions, and corrective surgery for scoliosis. His best-corrected visual acuity (BCVA) was 20/400 in both eyes. Ocular examination showed a normal anterior segment, while fundus examination revealed pale disc, vessel attenuation, papillomacular bundle loss, and mild peripheral pigmentary changes (Fig. 1A). Optical coherence tomography (OCT) of the macula showed retinal thinning, ellipsoid zone disruption, and intraretinal cystoid changes in both eyes (Fig. 1B). Fundus autofluorescence (FAF) revealed a C-shaped pattern with a double hyper-autofluorescent (AF) ring in both eyes (Fig. 1C). The full-field electroretinogram disclosed cone-rod dystrophy, and the pattern of ERG showed extinguished P50 and N95, indicating dysfunction in both photoreceptors and retinal ganglion cells (RGCs). (Fig. 1D).

We further performed genetic testing under the impression of inherited eye disease, and whole-exon sequencing (WES) detected a heterozygous *SSBP1* missense variant (NM_003143.3) c.113G > A, (p.Arg38Gln), leading to the diagnosis of OPA13 with retinal and foveal abnormalities. This variant has been reported in several OPA13 individuals with an autosomal dominant inheritance pattern and has been classified as pathogenic in ClinVar (Variation ID: 977502). Sanger sequencing of the patient confirmed the *SSBP1* missense variant of the patient (Fig. 1E).

**Fig. 2:**
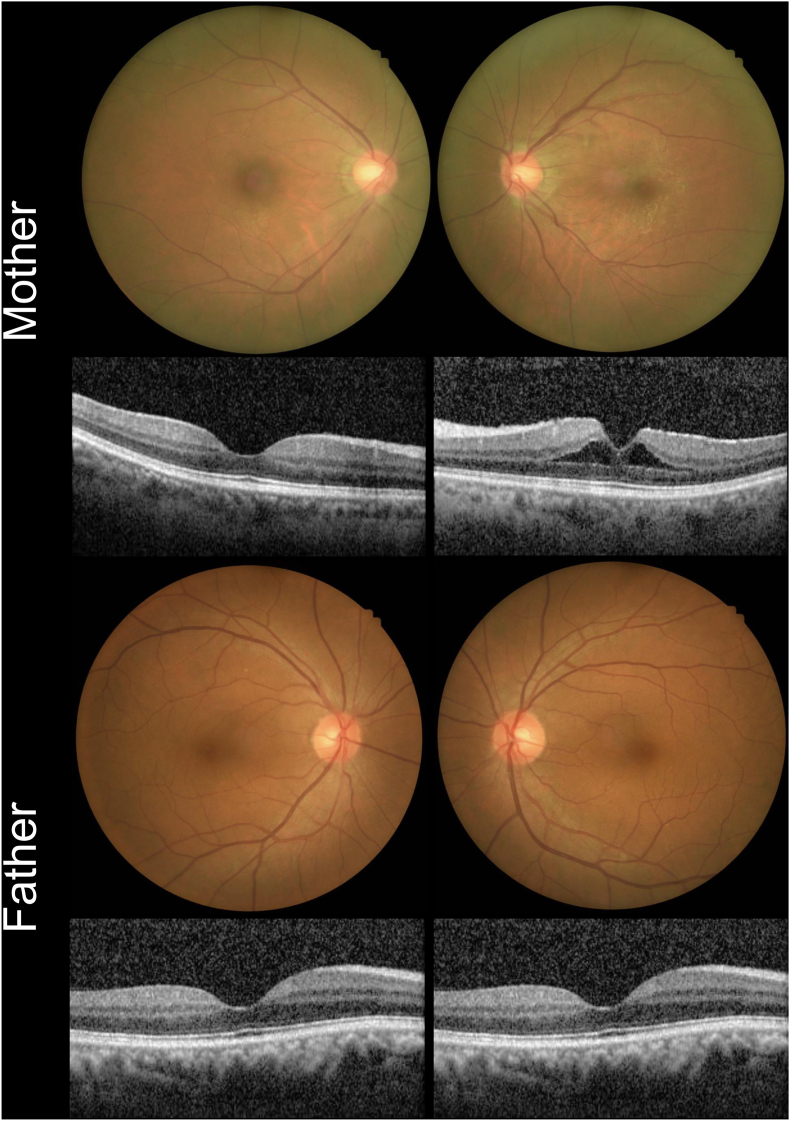
Color fundus photography and OCT of both parents: the mother had an epiretinal membrane and cystoid macular edema in the left eye, with no other abnormalities OCT, Optical coherence tomography. (For interpretation of the references to colour in this figure legend, the reader is referred to the Web version of this article.)

The patient reported no family history of visual disorders. His 54-year-old father had a BCVA of 20/50 in the right eye and 20/28 in the left, while his 52-year-old mother had a BCVA of 20/20 in both eyes, with an epiretinal membrane and cystoid macular edema in the left eye. All the other examinations were considered normal for both parents (Fig. 2). WES and Sanger sequencing for both parents revealed no *SSBP1* variants at the same locus, except for a c.224T > C (p.Leu75Pro) variant from his mother, which is genetically insignificant. Therefore, the patient was confirmed to have a de novo *SSBP1* gene variant.

## 3 Discussion

Our case report describes a 19-year-old male OPA13 patient with a heterozygous *SSBP1* missense variant, featuring a double C-shaped AF ring on fundus FAF imaging. FAF imaging in retinitis pigmentosa (RP) typically reveals a single hyper-AF ring, where photoreceptor layers within the ring's inner border are generally preserved, while outer areas show thinning or loss of the outer nuclear layer.^7^ However, our patient exhibited a double C-shaped AF ring, which differs from the typical single ring seen in most RP cases. While this specific pattern has not been widely reported, it may represent a potential feature associated with *SSBP1*-related retinopathy, including OPA13. However, further cases are needed to determine its diagnostic value. Although sporadic cases of OPA13 are not uncommon, comprehensive parental testing is not always performed, and low-level mosaicism may be missed by conventional sequencing methods. In our case, genetic testing via WES and Sanger sequencing in both the patient and his parents confirmed the presence of a de novo variant in the *SSBP1* gene. Our findings highlight the utility of sensitive genetic approaches for accurate diagnosis and counseling.

The patterns of retinal degeneration in inherited retinal diseases are not static and evolve over time. It is possible that the C-shaped image does not manifest in all cases and may be absent in the early or advanced stages of disease progression. Additionally, this pattern might resemble an early-stage presentation in pericentral RP before a complete ring develops. While this feature was noted in our patient, additional studies are needed to determine its wider clinical relevance.

OPA13 represents a distinct type of dominant optic atrophy (DOA), a neuro-ophthalmic condition characterized by RGC degeneration, often associated with retinal degeneration and foveopathy, primarily due to variants in the *SSBP1* gene. Most DOA patients present with bilateral optic atrophy without photoreceptor involvement. The presence of optic atrophy with retinal and foveal abnormalities in this patient underscores the importance of considering OPA13 in the differential diagnosis of other DOA types. Additionally, genetic testing supports the diagnosis of OPA13 and may help anticipate systemic conditions such as sensorineural deafness and nephropathy.^2^ Although the patient did not present with hearing loss or renal failure, it is crucial to maintain close monitoring for potential systemic complications in individuals with pathogenic *SSBP1* variants.

In conclusion, this case report details a 19-year-old male with OPA13, showing a double C-shaped AF ring. In contrast to the typical single ring observed in RP, this distinct pattern may aid in the diagnosis of OPA13 and may serve as a useful phenotypic indicator in certain cases, although further research is required. Genetic testing identified our case as a de novo *SSBP1* variant, linking this variant to the newly found retinal feature and highlighting the importance of considering OPA13 in similar cases.

## CRediT authorship contribution statement

**Hsin Hsu:** Writing – original draft, Formal analysis. **Chunya Kang:** Writing – review & editing. **Eugene Yu-Chuan Kang:** Supervision, Investigation, Conceptualization. **Nan-Kai Wang:** Supervision, Resources, Methodology.

## Patient consent

Consent to publish this case report has been obtained from the patient in writing.

## Authorship

All authors attest that they fulfil the current ICMJE criteria for authorship.

## Funding

This research was supported by 10.13039/100012553Chang Gung Memorial Hospital, Taiwan (CMRPG3N1001), the 10.13039/100020595National Science and Technology Council, Taiwan (NSTC 113-2314-B-182A-150-MY3) and 10.13039/501100002836Chang Gung University, Taiwan (UARPD1N0031 and UARPD1P0261).

## Declaration of competing interest

The authors declare that they have no known competing financial interests or personal relationships that could have appeared to influence the work reported in this paper.
